# The effects of cold shock on freshwater fish larvae and early-stage juveniles: implications for river management

**DOI:** 10.1093/conphys/coaa092

**Published:** 2020-10-13

**Authors:** Laura E Michie, Jason D Thiem, Craig A Boys, Simon M Mitrovic

**Affiliations:** 1 School of Life Sciences, University of Technology Sydney, 15 Broadway, Ultimo, New South Wales, 2007, Australia; 2 NSW Department of Primary Industries, Narrandera Fisheries Centre, 70 Buckingbong Road, Narrandera, New South Wales, 2700, Australia; 3 NSW Department of Primary Industries, Port Stephens Fisheries Centre, Taylors Beach Road, Taylors Beach, New South Wales, 2316, Australia

**Keywords:** Cold shock, freshwater fish, ontogeny, temperature

## Abstract

Temperature is essential to the maintenance of optimal physiological functioning in aquatic organisms. Fish can manage natural fluctuations in temperature; however, in freshwater ecosystems acute and rapid temperature changes can originate from sources such as large dams and industrial effluents. These rapid temperature changes may induce several physiological and behavioural responses that can result in lethal and sub-lethal consequences. The present study assessed immediate sub-lethal and short-term (10 days) lethal responses of three species of Australian freshwater fish larvae and early-stage juveniles to a range of different ‘field-relevant’ cold shocks (−4, −6, −8 and −10°C). Murray cod (*Maccullochella peelii*), silver perch (*Bidyanus bidyanus*) and golden perch (*Macquaria ambigua*) were tested at two age groups to elucidate the interaction between ontogeny and sensitivity to cold shock. Cold shock caused mortality and reductions in swimming ability (time to exhaustion and lines crossed) in all species of fish at both age groups. Sensitivity was correlated to the magnitude of cold shock; a 10°C drop in temperature caused the highest mortalities. Ontogeny interacted with the severity of cold shock; the younger fish experienced higher mortalities and greater impairment to swimming ability. This study demonstrates the potential lethal and sub-lethal impacts of cold shock on freshwater fish at a critical life-history stage. Understanding the impacts of cold shock will aid management of freshwater ecosystems for the benefit of fish populations, with the current study identifying critical life stages to be considered in remediation and guiding thresholds necessary to reduce the impact of cold shock on native fish populations.

## Teaser

Rapid reductions in water temperature can originate in freshwater ecosystems from the operation of dams. We demonstrate that these ‘cold shocks’ can cause mortality and reduce swimming ability in three species of Australian freshwater fish larvae and early-stage juveniles, with younger fish being more sensitive to cold shock.

## Introduction

Temperature plays an important role in the regulation of physiological, metabolic and behavioural processes of fish, supporting optimal rates of reproduction, growth, metabolism and locomotion (Beitinger *et al.*, 2000, Brett, 1971, Clarkson and Childs, 2000, Jobling, 1995, Jobling, 1997). Optimal thermal ranges for fish are species-specific and are formed as a result of genetic and thermal histories and may vary between life-history stages (Beitinger *et al.*, 2000, Donaldson *et al.*, 2008, Jobling, 1995). When exposed to thermal stress, generally increased mortality occurs in younger and smaller conspecifics (Berry, 1988, Sogard, 1997). Typically fish can manage natural variations to the thermal regime including diel and seasonal events; however, acute and rapid changes in temperature may cause direct mortality or induce sub-lethal physiological and behavioural responses (Donaldson *et al.*, 2008). The effect of cold shock exposure on fish is stronger as the rate and magnitude of temperature change are increased and as temperatures approaches the limits of species-specific or ontogenetic thermal tolerance ranges (Donaldson *et al.*, 2008).

Thermal shock can instigate a range of stress responses in fish (Donaldson *et al.*, 2008) and has been linked to fish kills in a number of species (Ash *et al.*, 1974, Bohnsack, 1983, Cyrus and McLean, 1996, Szekeres *et al.*, 2014, Young and Gibson, 1973). Recent studies on cold shock responses in fish have shifted from lethal responses to sub-lethal responses (Donaldson *et al.*, 2008), with much focus on fluctuating levels of hormones as a measure of physiological stress (Barton and Peter, 1982, Datta *et al.*, 2002, Donaldson *et al.*, 2008, Inoue *et al.*, 2008, Tanck *et al.*, 2000). Although it is widely acknowledged that cold shock causes stress in fish, few studies assess how this physiological stress is manifested through behavioural consequences that may ultimately affect survival and fitness (Donaldson *et al.*, 2008, Szekeres *et al.*, 2016). Understanding the link between physiological stress and behavioural responses in fish is essential in determining how populations may be affected by environmental stressors. Early investigations of the effects of cold shock on fish observed that fish entered a period of ‘cold coma’ where there was a noticeable loss of swimming ability after exposure to cold shock although there was no quantifiable measure of impaired swimming ability (Berry, 1988, Clarkson and Childs, 2000). More recent analysis has sought to effectively quantify reflex impairment, metabolic performance and swimming ability in juvenile and adult fish after exposure to cold shock stress to better understand subsequent ecological consequences (Parisi *et al.*, 2020, Rodgers *et al.*, 2014, Szekeres *et al.*, 2014).

Rapid reductions in water temperature that may instigate cold shock responses in fish can originate from a range of natural or anthropogenic sources. Natural sources may include rapid changes in diel temperatures, variation in solar-heat exposure in shallow waters, the presence of thermoclines and mixing events in lentic systems (Donaldson *et al.*, 2008). Anthropogenic sources may include industrial effluents (Coutant, 1977), ocean thermal energy conversion facilities (Lamadrid-Rose and Boehlert, 1988) and water releases from large dams (Michie *et al.*, 2020, Ryan and Preece, 2003). Dams are known to alter natural river thermal regimes, breaking the natural river continuum and causing thermal pollution in downstream ecosystems (Lugg and Copeland, 2014, Ward and Stanford, 1983). Thermally stratified dams can have discernible temperature differences between surface waters and bottom waters of up to 16°C (Bonnet *et al.*, 2000, Hart and Sherman, 1996, Lugg and Copeland, 2014, Preece and Jones, 2002, Sherman, 2000). When water releases originate from different depths within the dam and are interchanged rapidly (through the use of multi-level outlets or spillway and bottom-outlet releases), acute and rapid temperature changes can occur in downstream stretches of river (Gaillard, 1984, Michie *et al.*, 2020, Ryan and Preece, 2003). Additionally, dams can create conditions where there are discernible differences in water temperatures between the main channel and its tributaries; this can occur when the regulated main channel is affected by cold water pollution and its tributaries are not. As a result, fish can be exposed to acute temperature changes when migrating between the thermally isolated channels (Clarkson and Childs, 2000, Ryan and Preece, 2003). The potential for thermal shock in freshwater ecosystems is often overlooked in water management plans (Donaldson *et al.*, 2008, Ryan and Preece, 2003). Considering the prevalence of large dams and their continued construction in developing regions (Lehner *et al.*, 2011, Winemiller *et al.*, 2016) and the increased potential for thermal variability in aquatic ecosystems under future climate change (Szekeres *et al.*, 2016), understanding the effect these structures can have upon fish is essential for effective water resource management that supports anthropogenic and environmental needs.

The objective of this study was to assess the interactive effects of ontogeny and cold shock upon swimming ability, and immediate and delayed mortality of three species of Australian freshwater fish; Murray cod (*Maccullochella peelii*), silver perch (*Bidyanus bidyanus*) and golden perch (*Macquaria ambigua*). These species were selected as they have historically suffered population declines since the onset of river regulation and they have a large distribution within the Murray-Darling Basin of Australia, a region identified for its high potential for cold shock due to the number of large dams, its warm climate and warm-water adapted fish species (Gehrke *et al.*, 1995, Mallen-Cooper, 1993, Ryan and Preece, 2003). To quantify the ecologically relevant consequences of rapid temperature shifts on fish that may affect fitness, measures of swimming ability were tested after exposures to a range of cold shocks that may occur in river ecosystems resulting from operation of dam infrastructure. We predicted that large magnitude cold shocks (−10 and −8°C) would result in high rates of immediate and delayed mortalities, but moderate cold shocks (−6 and −4°C) would instigate sub-lethal responses in fish that could affect individual fitness. We also predicted that ontogeny would affect the magnitude of the response with younger fish being more sensitive to cold shock exposure due to their expected narrower thermal tolerance range. Understanding the responses of larval fish to acute changes in water temperature that can be experienced in rivers is essential for managing the health and persistence of native fish populations.

## Methods

Murray cod, silver perch and golden perch larvae and early-stage juveniles were acquired from a government hatchery (Department of Primary Industries (DPI) Narrandera Fisheries Centre), where fish were sourced from internal (Murray cod) and external (silver perch and golden perch) ponds determined by standard hatchery practices required for the optimal raising conditions for each species. Fish were selected from mixed breeding pairs from brood-stock sourced from the Murray-Darling Basin, and trials were run sequentially to accommodate for variation in the specific time of breeding between the different species and the age ranges sampled. Fish were sampled at two age groups ~23 days apart; young (16–18 days post hatch (dph)) and old (Murray cod: 37–38 dph, silver perch and golden perch; 40–44 dph). Fish were transferred to glass holding aquaria (70 L) containing aerated bore water and were left to adjust overnight to laboratory conditions with a 12:12 hour light cycle and ambient room temperature of 23°C, an appropriate natural temperature for larvae of all three species (King *et al.*, 2016). Fish were held in aerated aquaria for a maximum of two nights. To ensure feeding did not interact with energy levels during the standardized chase to exhaustion assessments, they were only fed at night after the assessments were conducted; they were fed to satiation on a diet of Artemia (*Artemia franciscana*), which were hatched onsite. Parameters of dissolved oxygen, pH and conductivity were assessed daily prior to cleaning tanks of excess food and conducting an ~30% water exchange.

### Cold shock trials

Experimental aquaria (70 L) were maintained at test temperatures of 13, 15, 17, 19 and 23°C that were temperature controlled by water chillers (HC-300A Hailea, China). Temperatures were selected to cover a range of cold shock exposures that may be present in Australian freshwater ecosystems (Michie *et al.*, 2020, Ryan and Preece, 2003). The fish were transferred individually with nets from the holding aquaria (23°C) to the experimental aquaria where they were exposed to test temperatures of 13, 15, 17, 19 and 23°C, representing cold shock exposures of −10, −8, −6, −4 and 0°C. Oxygen levels in all trials were measured intermittently and maintained between 8.00–10.00 mg L^−1^. A mean sample size of 15 fish was exposed to each treatment. After exposure to the cold shock treatments, any mortalities that occurred in a 2-min period were recorded as immediate mortalities. After 2 min, surviving fish were sampled in behavioural impairment tests to determine the effect of cold shock upon swimming ability and speed. Fish were sampled over 2 days (per species and age group) and were randomly assigned to the cold shock exposures, and the sampling order of the exposures was randomized over the two sampling days.

### Standardized chase to exhaustion

Individual fish were transferred to tanks where they were exposed to the cold shock exposures (−10, −8, −6, −4 and 0°C). After 2 min, surviving fish were then transferred to an annular swim flume (a circular tank divided into four equal quadrants with the centre blocked to form a swimming ring) containing 400 mL of water at the exposure temperature (Portz, 2007). The swimming assessments were conducted with both a handled control (HC) and a control (C). The 0°C treatment represented the handled control; fish were treated in the same manner as the cold shock treatments. In the control, fish were transferred directly to the annular swim flume from the holding tank. A standardized chase to exhaustion was conducted. Fish were chased around the annular swim flume by hand, and time to exhaustion was measured in seconds (s) when no escape response was exhibited to three consecutive tail probes. Within the first 30 s of the standardized chase to exhaustion, the number of lines (quadrants) crossed by the fish was counted. Similar methods were used to assess behavioural impairment in adult bonefish when exposed to cold shock (Szekeres *et al.*, 2014) and in adult checkered puffers (*Sphoeroides testudineus*) to assess the use of radio tags on swimming ability (Thiem *et al.*, 2013).

### Prolonged-exposure mortality

We investigated how prolonged exposure to rapid reductions in water temperature influenced mortality over a 10-day period. Fish sampled in this assessment were not included in the standardized chase to exhaustion. Fish were transferred to experimental tanks and exposed to cold shock exposures of −10, −8, −6, −4 and 0°C; they were then held at these temperatures for 10 days. This maintained suppression in water temperatures commonly occurs in areas where fish kills are caused by cold shock (Szekeres *et al.*, 2014) and would allow us to determine if fish are likely to experience delayed mortality under these conditions. Mean sample size of fish exposed to each treatment varied between species (MC = 14, SP = 30, GP = 29), and fish were divided between four replicate tanks. Murray cod were not tested for extended mortality at −4°C due to resource limitations. Mortality was measured hourly for the first 12 h post exposure and then was assessed at 24-h intervals for the duration of the experiment. After exposure, fish were fed three times daily to satiation on a diet of Artemia. Tanks were cleaned daily, with an ~30% water exchange.

### Data analysis

Immediate mortality was assessed as the percentage of fish that experienced mortality in the two-minute exposure to the cold shock treatments and a chi-square test assessed independence. Log-rank survival analysis was used to test whether the cold shock exposures caused delayed mortality; this was then plotted for each age group and species using a Kaplan–Meier survival estimate.

A parametric two-way analysis of variance (ANOVA) was used to test for an effect and interaction of cold shock and ontogeny (fixed effects) on time to exhaustion and lines crossed (response variables—separate models). Where assumptions for parametric analysis were not met, a log transformation of the data was performed. Differences between treatments for the standardized chase to exhaustion assessments were assessed with Tukey’s post hoc analysis and a Bonferroni correction was undertaken to account for the high number of multiple comparisons involved in the analysis and reduce pair-wise error rates. All statistical analysis were conducted in R version 3.5.1 (R Core Team, 2019) with a minimum significance level of *α* < 0.05.

**Figure 1:**
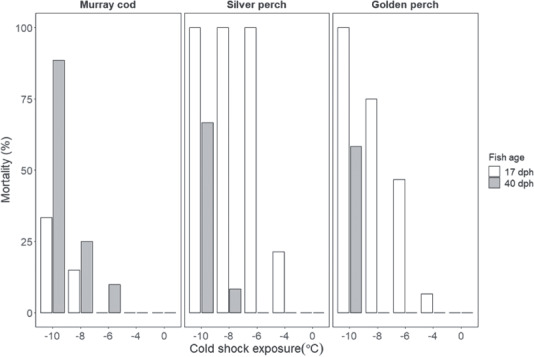
Mortality (%) of Murray cod, silver perch and golden perch occurring within 2 min of exposure to cold shock treatments (−10, −8, −6, −4 and 0°C) from an initial temperature of 23°C. Two age groups are assessed; old (40 ± 3 dph) and young (17 ± 1 dph)

## Results

### Immediate mortality

Exposure to rapid reductions in water temperature caused immediate mortality in Murray cod (*χ*^2^ = 403.69, df = 9, *P* < 0.001), silver perch (*χ*^2^ = 485.78, df = 9, *P* < 0.001) and golden perch (*χ*^2^ = 454.61, df = 9, *P* < 0.001). Generally, mortality increased as the magnitude of cold shock was more extreme; however, there were differences between species and within age groups. Silver perch and golden perch at 17 dph were most sensitive to immediate mortality than Murray cod at the same age (Fig. 1). In silver perch and golden perch, at 17 dph some mortality occurred with as little as a 4°C cold shock, whereas Murray cod mortality occurred at 17 dph with cold shocks at >8°C. Furthermore, mortality rates were higher in silver perch and golden perch than Murray cod; 100% mortality occurred in silver perch (17 dph) at the three highest exposures (−10, −8 and −6°C) and in golden perch (17 dph) at the highest exposure (−10°C), whereas Murray cod mortality rates at 17 dph were ~33% at the highest magnitude cold shock (10°C). In the older age class (40 dph), Murray cod susceptibility to cold shock was more consistent with the two perch species; all experienced mortalities at the highest magnitude cold shock (10°C).

### Prolonged-exposure mortality

**Figure 2:**
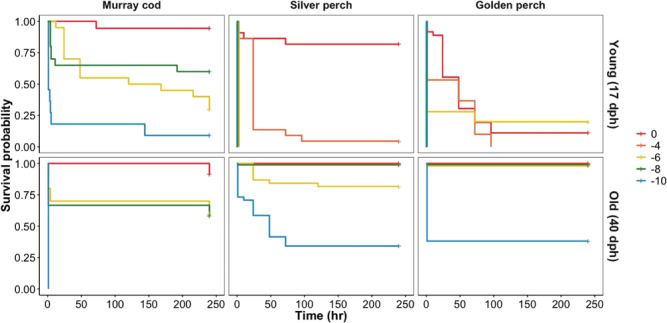
Survival analysis of Murray cod, silver perch and golden perch over 10 days after exposure to cold shock treatments (−10, −8, −6, −4 and 0°C) from an initial temperature of 23°C. Two age groups are assessed; old (40 ± 3 dph) and young (17 ± 1 dph)

Exposure to rapid reductions in water temperature resulted in delayed mortality (Fig. 2). In the youngest age group, treatment affected survival probability of Murray cod (log-rank survival analysis: *χ*^2^ = 42.9, df = 3, *P* < 0.001), silver perch (log-rank survival analysis: *χ*^2^ = 143, df = 4, *P* < 0.001) and golden perch (log-rank survival analysis: *χ*^2^ = 146, df = 4, *P* < 0.001). Murray cod experienced delayed mortalities when water temperature was reduced by 6, 8 and 10°C. Silver perch exhibited delayed mortality in fish exposed to a 4°C drop with <90% surviving after 10 days. Golden perch exhibited delayed mortalities in all treatments, including the control. In the oldest age group (42 dph), treatment affected survival probability of Murray cod (log-rank survival analysis: *χ*^2^ = 25.1, df = 3, *P* < 0.001), silver perch (log-rank survival analysis: *χ*^2^ = 182, df = 4, *P* < 0.001) and golden perch (log-rank survival analysis: *χ*^2^ = 166, df = 4, *P* < 0.001). Most mortalities occurred immediately after the cold shock, except for silver perch with delayed mortalities occurring particularly after a 10°C reduction in temperature.

### Standardized chase to exhaustion

**Figure 3:**
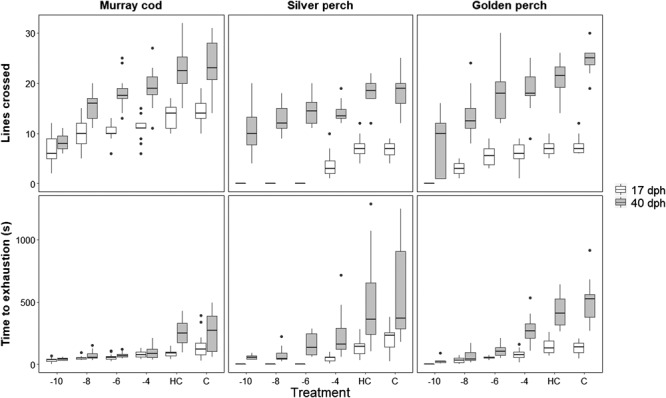
Assessment of lines crossed (within 30 s) and time to exhaustion (s) of Murray cod, silver perch and golden perch after exposure to cold shock treatments (−10, −8, −6, −4°C) from an initial temperature of 23°C. Assessment commenced 2 min after exposure to the cold shock treatments. A handled control (HC) and control (C) were used to determine effect of handling. Two age groups are assessed; old (40 ± 3 dph) and young (17 ± 1 dph)

Exposure to rapid reductions in temperature reduced swimming ability in terms of number of lines crossed and time to exhaustion in fish of both ages (Fig. 3). Treatment significantly affected lines crossed by Murray cod (*F_5, 193_* = 46.580, *P* < 0.001), silver perch (*F_5, 90_* = 6.349, *P* < 0.001) and golden perch (*F_5, 108_* = 35.15, *P* < 0.001). Number of lines crossed and time to exhaustion could not be tested in 17 dph golden perch exposed to a 10°C cold shock and 17 dph silver perch exposed to a 10, 8 and 6°C cold shock as all fish experienced mortality prior to being tested in the standardized chase to exhaustion. Although there was overlap in subsequent treatments, the number of lines crossed in the first 30 s of the chase to exhaustion was maximized in the controls and reduced at higher magnitude treatments. Time to exhaustion was affected by the cold shock treatments in Murray cod (*F_4, 156_* = 43.798, *P* < 0.001), silver perch (*F_5, 90_* = 13.670, *P* < 0.001) and golden perch (*F_5, 109_* = 41.258, *P* < 0.001). Murray cod and silver perch that were exposed to cold shock fatigued quicker than controls; however, there was no difference in time to exhaustion between the different cold shock exposures (see supplementary data). In golden perch, fish exposed to cold shock fatigued earlier than fish exposed to a 4°C cold shock and controls; however, there was no difference in time to exhaustion in fish that had been exposed to 10, 8 and 6°C cold shocks (see supplementary data).

Age affected swimming ability in terms of lines crossed (LC) and time to exhaustion (TTE) in all species; Murray cod (LC: *F_1, 193_* = 300.520, *P* < 0.001, TTE: *F_1, 156_* = 16.484, *P* < 0.001), silver perch (LC: *F_1, 90_* = 255.594, *P* < 0.001, TTE: *F_1. 90_* = 57.731, *P* < 0.001) and golden perch (LC: *F_5, 108_* = 319.35, *P* < 0.001, TTE: *F_1, 109_* = 115.852, *P* < 0.001). The youngest age group swam across fewer lines and fatigued faster. Handling did not affect the number of lines crossed or time to exhaustion in any species (see supplementary data).

## Discussion

Survival of fish is highly variable through larval and juvenile life-history stages and plays an essential role in determining fish population dynamics (Sogard, 1997). We demonstrated that acute decreases in water temperature can induce cold shock responses in three species of Australian freshwater fish larvae and early-stage juveniles resulting in physiological impairment, as well as accentuating mortality rates at a sensitive life-history stage. Acute thermal reductions have caused mortality of fish species in freshwater, estuarine and marine ecosystems (Ash *et al.*, 1974, Szekeres *et al.*, 2014). Mechanical failure of a power plant situated on Lake Wabamun, Alberta Canada, caused cessation of warm water discharge that had attracted fish to the power plant discharge channel. There was a resultant cold shock within the channel, causing a large fish kill that included spottail shiners (*Notropis hudsonius*) and northern pike (*Esox lucius*) (Ash *et al.*, 1974)*.* In 2010, a fish kill occurred in Florida that was estimated to include hundreds of thousands of fish including bonefish (*Albula vulpes*), Atlantic tarpon (*Megalops atlanticus*) and common snook (*Centropomus undecimalis*); the fish kill occurred after water temperatures declined by 11.2°C following an extended cold weather event in the region (Szekeres *et al.*, 2014). Fish kills in the region of similar nature have occurred in the past (Bohnsack, 1983). A climatic cold spell on the eastern coast of South Africa caused a fish kill of an estimated 250 000 fish comprising of at least 21 species (Cyrus and McLean, 1996). Estimates of the numbers of fish affected in fish kills are often misrepresented, largely due to the potential for fish to sink within the water column or to be transported through river flows, tides or currents (La and Cooke, 2011, Young and Gibson, 1973). Considering that we observed delayed mortalities after cold shock exposure in Murray cod and silver perch larvae, we highlight the potential for an underrepresentation of fish counted within fish kill events.

Mortality rates in silver perch, Murray cod and golden perch were higher at higher magnitude cold shock exposures; mortality was highest at a cold shock of 10°C. This observation is consistent with experimental trials that link the severity of cold shock to mortality in several other species of fish. For example, mortality of 14-day-old Colorado swordfish (*Ptychocheilus lucius*) larvae was higher after exposure to a 15°C cold shock compared to 5 and 10°C cold shocks (Berry, 1988). Similarly, juvenile striped mullet (*Mugil cephalus*) had significant mortality after a 15°C cold shock but not after 5 and 10°C cold shocks (Lamadrid-Rose and Boehlert, 1988). Recently fertilized eggs of mahimahi (*Coryphaena hippurus*) experienced a mortality rate of 100% when exposed to cold shocks of 10 and 15°C; however, mortality was significantly reduced (20%) at a lower magnitude 5°C cold shock (Lamadrid-Rose and Boehlert, 1988). Similarly, in eggs of manini (*Acanthurus triostegus*) mortality increased with increasing magnitude of cold shock (Lamadrid-Rose and Boehlert, 1988). As cold shocks of higher magnitude increase the likelihood of mortality occurring in fish, we demonstrate that reducing the potential for cold shock of large magnitudes that currently exists in large rivers (Michie *et al.*, 2020) would be a useful management tool for native freshwater fish.

Reduced swimming speed limits and endurance in fish can directly impact predator–prey interactions, foraging behaviour and the ability to complete migrations and traverse fish passage structures. As such, sub-lethal responses to cold shock in freshwater ecosystems can eventuate in ecological consequences for fish that may prove lethal (Domenici and Blake, 1997, Green and Fisher, 2004, Videler and Wardle, 1991, Wolter and Arlinghaus, 2003). Cold shock exposure reduced swimming ability in three species of fish; as the magnitude of cold shock increased, fish experienced greater impairment to their swimming speed, while any exposure to cold shock reduced time to exhaustion. It is widely documented that chronic reductions in water temperature reduce fish swimming ability (Claireaux *et al.*, 2006, Lyon *et al.*, 2008, Myrick and Cech, 2000, Starrs *et al.*, 2011, Ward *et al.*, 2002), but the understanding of the effects of acute temperature cold shock is much more limited. Similar to our observations, after a 10°C cold shock juvenile silver perch exhibited reduced sprint and critical swimming speed (Parisi *et al.*, 2020) and juvenile empire gudgeon (*Hypseleotris compressa*) and Australian bass (*Percalates novemaculeata*) exhibited reduced critical swimming speeds (Rodgers *et al.*, 2014). Although with similar results, most other research is mostly based on behavioural observation rather than quantification (Berry, 1988, Clarkson and Childs, 2000). After cold shock exposure, reduced activity levels were noted in larval and juvenile fish native to the Colorado River Basin; razorback Sucker (*Xyrauchen texanus*), flannelmouth sucker (*Catostomus latipinnis*), humpback chub (*Gila cypha*) (Clarkson and Childs, 2000) and Colorado pikeminnow (*Ptychocheilus lucius*) (Berry, 1988). At the highest magnitude cold shocks (>10°C), the impact on swimming ability was strongest, with fish entering a state of ‘cold coma’ (Berry, 1988, Clarkson and Childs, 2000).

Cold coma is a physiological state that relates to osmoregulatory dysfunction where fish exhibit a loss of equilibrium and lack the ability to maintain their position within the water column (Berry, 1988, Clarkson and Childs, 2000, Pitkow, 1960). Adult bonefish (*Albula vulpes*) exposed to a 14°C cold shock also exhibited loss of equilibrium (Szekeres *et al.*, 2014). Berry (1988) notes that larval and juvenile fish that recovered from cold coma in experimental trials would not be likely to do so under natural conditions, where additional stressors such as high flow rates, irregular instream habitat and predators may interfere with survival. Reduction in swimming ability is likely to manifest in increased predation; this has been observed in juvenile channel catfish (*Ictalurus punctatus*) and largemouth bass (*Micropterus salmoides*) by adult largemouth bass after exposure to cold shock, with the rate of predation increasing with increasing magnitude of the cold shock exposure (Coutant *et al.*, 1974). Similarly, predation on bluegill (*Lepomis macrochirus*) was increased after cold shock exposure (Wolters and Coutant, 1976). Both these studies analysed predation rates with unstressed predators; although larger fish are less susceptible to acute thermal stress, future analysis should focus on interactions where both prey and predators are stressed to better simulate natural conditions.

Young fish were more susceptible to cold shock than older fish. The only exception to this was in assessment of immediate mortality in Murray cod after cold shock exposure, where older fish were more likely to experience mortality. Considering delayed mortalities of Murray cod over a 10-day period, the youngest age group experienced delayed mortalities where the older age group did not and as a result there was a higher cumulative mortality within the youngest age group. This suggests that physiological plasticity is stronger in older fish and leaves them more equipped to cope with prolonged exposure to thermal stress. Younger Colorado pikeminnow larvae were more sensitive to cold shock than older conspecifics; 14 days post hatch (dph) larvae experienced significant mortalities after a 15°C cold shock whereas 40-dph juveniles did not (Berry, 1988). When exposed to smaller magnitude cold shocks (10 and 5°C), 14-dph larvae exhibited reduced movement whereas 40-dph juveniles were not affected by the same exposure. Similarly, ontogeny interacted with cold shock exposure in razorback sucker, flannelmouth sucker and humpback chub (Clarkson and Childs, 2000)*.* When exposed to a 10°C cold shock the youngest age group (5–9 dph) was the most sensitive across all species and entered ‘cold coma’; older age groups (11–15 and 42–43 dph) either experienced no behavioural changes to the stress or, in the case of humpback chub, recovered at a faster rate (Clarkson and Childs, 2000). When exposed to cold shock, ontogeny of manini and mahimahi eggs interacted with sensitivity to the stress; subsequent mortalities and deformities were higher in earlier stage eggs (Lamadrid-Rose and Boehlert, 1988). Small body size and reduced developmental stage increases sensitivity of fish to environmental stressors such as temperature (Sogard, 1997). We demonstrated that even small variation in ontogeny (23 days) in fish can greatly affect the magnitude of their response and their susceptibility to acute thermal stress.

Fish can be subject to cold shock in freshwater ecosystems when large fluctuations in flow originate from upstream impoundments (Ryan and Preece, 2003), when varied dam release mechanisms are used interchangeably at large stratified dams (Michie *et al.*, 2020), and when fish migrate between main-channels that are affected by cold water pollution and thermally isolated tributaries (Clarkson and Childs, 2000, Koehn, 2001). In Australia, these potential cold shocks could range in magnitude of up to 10°C in the case of altered dam management (Michie *et al.*, 2020, Ryan and Preece, 2003) or as much as 15°C during fish migrations across tributary/main-channel boundaries considering maximum potential cold water pollution (Lugg and Copeland, 2014). We demonstrate that cold shock that can occur in Australian freshwater ecosystems is likely to have a profound impact upon native fish communities through direct mortality and reduced swimming ability. Although sub-lethal, measures of swimming ability such as speed and fatigue have important ecological applications that may affect predator–prey interactions, migratory behaviour, foraging behaviour and the ability to traverse fish passage structures (Domenici and Blake, 1997, Videler and Wardle, 1991, Wolter and Arlinghaus, 2003).

Cold shock in Australian freshwater ecosystems is most likely to occur when stratification persists in dams and cold water pollution is most prolific; this occurs during summer months, which coincides with essential periods for Australian native fish breeding and larval development and is likely to affect fish at the ages we tested (Lugg and Copeland, 2014, Ryan *et al.*, 2001). The interaction between ontogeny and sensitivity to cold shock can guide management of potential sources of cold shock in aquatic ecosystems; delaying changes in the operation of dams that may cause cold shock could have significant benefits for downstream fish populations. Operational strategies that would reduce the rate of thermal change may also aid downstream fish populations (Burton *et al.*, 1979). When given the opportunity for acclimation, aquatic organisms are less sensitive to acute thermal changes (Seebacher *et al.*, 2015).

## Conclusion

Considering the widespread potential for cold shock in aquatic ecosystems from anthropogenic sources such as large dams, industrial effluents and ocean thermal energy conversion facilities (Coutant, 1977, Lamadrid-Rose and Boehlert, 1988, Michie *et al.*, 2020, Ryan and Preece, 2003) and the potential for cold shock to cause direct mortality or induce sub-lethal physiological and behavioural responses (Donaldson *et al.*, 2008), it is necessary to understand the impact of these operations on fish populations. We demonstrated that exposure to cold shock of a range of magnitudes (−10, −8, −6 and −4°C) can cause mortality and reduced swimming capabilities in fish. Small variation in ontogeny affected the susceptibility of Murray cod, silver perch and golden perch to acute thermal stress. Considering the global prevalence and the continued construction of large dams in developing regions (Lehner *et al.*, 2011, Winemiller *et al.*, 2016), understanding how these structures can instigate lethal and non-lethal responses in fish is essential for effective water resource management that supports anthropogenic and environmental needs.

## Funding

This work was supported by funding received from the NSW Department of Primary Industries, the Australian Wildlife Society, and the Fisheries Scientific Committee.

## Supplementary Material

Supplementary_Data_coaa092
