# Erratum to: The effects of cold shock on freshwater fish larvae and early-stage juveniles: implications for river management

**DOI:** 10.1093/conphys/coaa106

**Published:** 2020-11-05

**Authors:** Laura E Michie, Jason D Thiem, Craig A Boys, Simon M Mitrovic

**Affiliations:** 1 School of Life Sciences, University of Technology Sydney, PO Box 123, Broadway, New South Wales, 2007, Australia; 2 NSW Department of Primary Industries, Narrandera Fisheries Centre, PO Box 182, Narrandera, NSW, 2700, Australia; 3 NSW Department of Primary Industries, Port Stephens Fisheries Centre, Taylors Beach Road, Taylors Beach, New South Wales, 2316, Australia

Due to an error during the production, the version of this article that was first published contained errors within Figure 3. The figure has now been corrected. The publisher apologises for this error.

