# Supplementary material for: The effects of cold shock on freshwater fish larvae and early-stage juveniles: implications for river management
Source: Conserv Physiol. 2020 Oct 13;8(1):coaa092. doi: 10.1093/conphys/coaa092 (PMC7566967; doi:10.1093/conphys/coaa092)
Supplement: Supplementary_Data_coaa092 [file supplementary_data_coaa092.docx]

Supplementary table 1: Summary of Tukey’s post-hoc analysis (with Bonferroni correction) of the parametric two-way analysis of variance of lines crossed (within 30 seconds) and time to exhaustion (s) of Murray cod, silver perch and golden perch after exposure to cold shock treatments (-10°C, -8°C, -6°C, -4°C) from an initial temperature of 23°C. Assessment commenced two minutes after exposure to the cold shock treatments. A handled control (HC) and control (C) were used to determine effect of handling. Significant differences between treatments are indicated in bold.

| Species | Measure |  | -8⁰C | -6⁰C | -4⁰C | HC | C |
| --- | --- | --- | --- | --- | --- | --- | --- |
| Murray cod | Lines crossed | -10⁰C | **<0.001** | **<0.001** | **<0.001** | **<0.001** | **<0.001** |
|  |  | -8⁰C |  | 0.181 | **<0.001** | **<0.001** | **<0.001** |
|  |  | -6⁰C |  |  | 0.338 | **<0.001** | **<0.001** |
|  |  | -4⁰C |  |  |  | **<0.05** | **<0.01** |
|  |  | HC |  |  |  |  | 0.933 |
|  | Time to exhaustion | -10⁰C | 1.000 | 1.000 | 1.000 | **<0.001** | **<0.001** |
|  |  | -8⁰C |  | 1.000 | 1.000 | **<0.001** | **<0.001** |
|  |  | -6⁰C |  |  | 1.000 | **<0.001** | **<0.001** |
|  |  | -4⁰C |  |  |  | **<0.001** | **<0.001** |
|  |  | HC |  |  |  |  | 1.000 |
| Silver perch | Lines crossed | -10⁰C | 0.872 | 0.285 | 0.892 | 0.935 | 0.939 |
|  |  | -8⁰C |  | 0.743 | **<0.05** | 0.999 | 0.998 |
|  |  | -6⁰C |  |  | **<0.001** | 0.323 | 0.319 |
|  |  | -4⁰C |  |  |  | **<0.01** | **<0.01** |
|  |  | HC |  |  |  |  | 1.000 |
|  | Time to exhaustion | -10⁰C | 1.000 | 1.000 | 1.000 | **<0.05** | **<0.001** |
|  |  | -8⁰C |  | 1.000 | 0.949 | **<0.001** | **<0.001** |
|  |  | -6⁰C |  |  | 1.000 | **<0.05** | **<0.001** |
|  |  | -4⁰C |  |  |  | **<0.05** | **<0.05** |
|  |  | HC |  |  |  |  | 1.000 |
| Golden perch | Lines crossed | -10⁰C | **<0.001** | **<0.001** | **<0.001** | **<0.001** | **<0.001** |
|  |  | -8⁰C |  | 0.959 | 0.100 | 0.726 | 0.155 |
|  |  | -6⁰C |  |  | 0.972 | 0.993 | 0.546 |
|  |  | -4⁰C |  |  |  | 0.707 | 0.098 |
|  |  | HC |  |  |  |  | 0.824 |
|  | Time to exhaustion | -10⁰C | 1.000 | 1.000 | **<0.001** | **<0.001** | **<0.001** |
|  |  | -8⁰C |  | 1.000 | **<0.001** | **<0.001** | **<0.001** |
|  |  | -6⁰C |  |  | **<0.001** | **<0.001** | **<0.001** |
|  |  | -4⁰C |  |  |  | **<0.001** | **<0.001** |
|  |  | HC |  |  |  |  | 0.570 |
